# Purification, biochemical characterization and structural modelling of alkali-stable β-1,4-xylan xylanohydrolase from *Aspergillus fumigatus* R1 isolated from soil

**DOI:** 10.1186/s12896-016-0242-4

**Published:** 2016-02-04

**Authors:** Rehan Ahmed Deshmukh, Sharmili Jagtap, Madan Kumar Mandal, Suraj Kumar Mandal

**Affiliations:** Department of Microbiology, School Of Life-Sciences, Pondicherry University, R.V. Nagar, Kalapet, Puducherry 605014 India

**Keywords:** *Aspergillus fumigatus* R1, β-1,4-xylan xylanohydrolase, Purification, structural modelling, I-TASSER

## Abstract

**Background:**

*Aspergillus fumigatus* R1 produced xylanase under submerged fermentation which degrades the complex hemicelluloses contained in agricultural substrates. Xylanases have gained considerable attention because of their tremendous applications in industries. The purpose of our study was to purify xylanase and study its biochemical properties. We have predicted the secondary structure of purified xylanase and evaluated its active site residues and substrate binding sites based on the global and local structural similarity.

**Results:**

Various microorganisms were isolated from Puducherry soil and screened by Congo-red test. The best isolate was identified to be *Aspergillus fumigatus* R1. The production kinetics showed the highest xylanase production (208 IU/ml) by this organism in 96 h using 1 % rice bran as the only carbon source. The purification of extracellular xylanase was carried out by fractional ammonium sulphate precipitation (30–55 %), followed by extensive dialysis and Bio-Gel P-60 Gel-filtration chromatography. The enzyme was purified 58.10 folds with a specific activity of 38196.22 IU/mg. The biochemical characterization of the pure enzyme was carried out for its optimum pH and temperature (5.0 and 50^0^C), pH and temperature stability, molecular mass (Mr) (24.5 kDa) and pI (6.29). The complete sequence of protein was obtained by mass spectrometry analysis. Apparent *Km* and *Vmax* values of the xylanase for birchwood xylan were 11.66 mg/ml and 87.6 μmol min^−1^ mg^−1^ respectively.

**Conclusion:**

Purified xylanase was analyzed by mass-spectrometry which revealed 2 unique peptides. Xylanase under current study showed significant production using agricultural residues and a broad range of pH stability in the alkaline region. Xylanase produced by *Aspergillus fumigatus* R1 could serve as the enzyme of choice in industries.

## Background

Xylan, is a major structural polysaccharide in plant cells and the second most abundant polysaccharide in nature accounting for approximately one-third of all renewable organic carbon on earth [1] Hemicellulose is a complex of polymeric carbohydrates including xylan, xyloglucan, glucomannan, galactoglucomannan and arabinogalactan [2]. This together with cellulose and lignin constitute the major polymeric constituents of plant cell walls [3, 4]. Within the cell wall structure, all three constituents interact via covalent and non-covalent linkages with the xylan being found at the surface between lignin and cellulose where it is believed to be significant for fibre cohesion and plant cell wall integrity. Due to the heterogeneous and complex structure, the complete hydrolysis of xylan requires a number of enzymes which carry out its hydrolysis by a phenomenon known as cooperativity exhibited by endo-1,4-β-xylanase (EC 3.2.1.8), β-D-xylosidase (3.2.1.37) and a series of enzymes that hydrolyze the side chain groups [5]. Based on the mode of action and mechanism of catalysis xylanases have been assigned to glycoside hydrolase (GH) families 10 and 11 however some xylanases have also been categorized to families 5, 7, 8 and 43 [6].

Agricultural substrates contain hemicelluloses which have been globally generated. The use of agricultural residues in bioprocesses not only helps to reduce the pollution problems but also serves as the alternate substrate for the production of secondary metabolites [7]. Fungi such as *Aspergillus* spp. and *Trichoderma* spp. are of great significance as they produce high levels of xylanase than yeast and bacteria [8]. In this study, xylanase was produced using agricultural waste by *Aspergillus fumigatus* R1 which was isolated and identified in our laboratory. The xylanase was purified and its biochemical properties were studied to establish its relationship with the other characterized xylanases. Mass-spectrometric analysis reported that the purified xylanase is putative. Moreover, purified xylanase demonstrated significant biochemical characteristics which make it a suitable industrial enzyme.

## Results

### Identification and Phylogenetic analysis of the culture R1

**Fig. 1:**
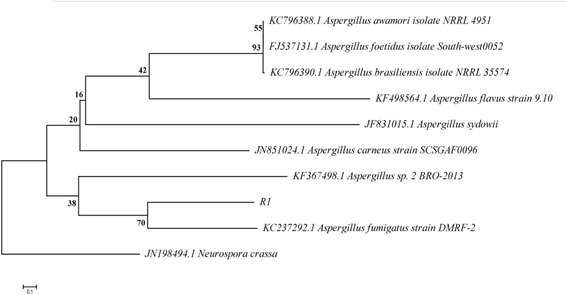
Phylogenetic tree of strain *Aspergillus fumigatus* strain R1 using the neighbor-joining method (MEGA 5.05) based on 18S rRNA gene sequences. Bootstrap values are represented as percentage at all branches. The scales indicate 0.1 substitutions per nucleotide and accession numbers of published strains of closely related strains are indicated

The xylanase-producing fungus was isolated on agar plate supplemented with the birchwood xylan as the sole source of the carbon at 37 ^0^C. The isolate was identified based on the highest xylanase activities as shown by the clear zone of hydrolysis when flooded with 0.1 % Cong-red stain. The best isolate was selected for further studies. Morphological characteristics indicated that the isolate is *Aspergillus.* Phylogenetic analysis of 18S rRNA gene sequence indicated that this strain shared 99 % sequence identity with 18S rRNA gene sequence of *Aspergillus fumigatus,* hence it was assigned as *Aspergillus fumigatus* strain R1 (Fig. 1).

### Xylanase production kinetics

**Fig. 2:**
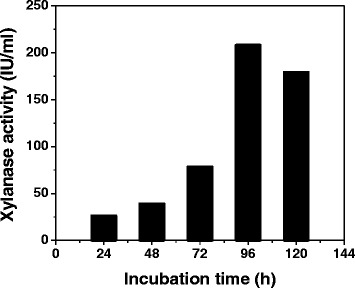
Kinetics of xylanase production *Aspergillus fumigatus* R1. After 96 h of incubation, xylanase activity was found to be highest and thereafter a decrease in production was reported

A submerged fermentation process was carried out with the optimized production medium as mentioned above. It was observed that *Aspergillus fumigatus* R1 produced xylanase and the highest yield reached 208 U/ml after 96 h of cultivation with rice bran as the only carbon source and decreased after 120 h (Fig. 2).

### Purification of Xylanase

**Fig. 3:**
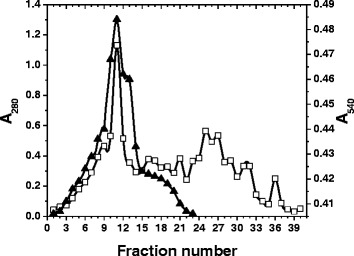
Bio-Gel P-60 gel-filtration elution profile of xylanase fractions, (▲) xylanase activity at A_540_, (□) A_280_

The xylanase activity increased with the increase of incubation time and reached the peak of activity about 208 U/ml after 96 h of incubation. So, the enzyme was harvested for the purification. *Aspergillus fumigatus* R1 xylanase was purified by fractional ammonium sulphate precipitation, dialysis and Bio-Gel P-60 gel-filtration chromatography. The result of the purification is summarized in Table 1. The crude supernatant was first subjected to 30–55 % fractional ammonium salt precipitation. The pellet obtained was then dissolved in minimum volume of phosphate buffer (pH 7) and subjected to extensive dialysis. After ammonium sulphate precipitation, the protein with a specific activity of 6300.28 U/mg was obtained whilst the specific activity of the enzyme preparation increased 9.04-fold. The dialysed enzyme sample was then subjected to Bio-Gel P-60 gel-filtration chromatography and the elution profile displayed more peaks of protein whilst a single elution peak of xylanase was obtained which indicated that xylanase of higher purity was achieved (Fig. 3). After the final step of purification, the enzyme was purified 58.10-fold with a specific activity of 38196.22 U/mg with a 3.43 % yield.

**Table 1:** Purification chart of xylanase from *Aspergillus fumigatus* R1

Purification step	Total activity^a^	Total Protein^b^	Specific activity	Purification fold	Yield
	(U)	(mg)	(U/mg)		(%)
Crude extract	176800	253.72	696.8	1	100
30-55 % (NH_4_)_2_SO_4_ Precipitation	43598	6.92	6300.28	9.04	24.6
Bio-Gel P-60 gel- Filtration	6073.2	0.159	38196.22	54.81	3.43

^a^xylanase activity was measured in 50 mM acetate buffer at 50^0^C for 30 min using 1.0 % (*w/v*) birchwood xylan as a substrate by the Dinitrosalicylic acid method [13]

^b^Measured by the method of Lowry et al. [34] using bovine serum albumin as a standard

### SDS-Polyacrylamide gel electrophoresis and activity analysis

SDS-PAGE of the purified xylanase was performed with linear (5–15 %) acrylamide gradient revealed single band when stained by silver staining method (supplementary data). The relative molecular mass of xylanase was found to be approximately 25 kDa. Wong et al. [9] illustrated a conserved relationship between molecular weight and p*I* of multiple xylanases from various microorganisms. For instance they are classified as low MW (16–22 kDa)/ basic (p*I,* 8.3–10.0) xylanases and high MW (43–50 kDa)/ acidic (p*I*, 3.6-4.5) xylanases. However, this system does not classify all xylanases from different microorganisms satisfactorily. The pure xylanase exhibited relatively clear activity under nondenaturating conditions using 2 % birchwood xylan in 12 % (*w/v*) acrylamide in gels. The activity was detected by Congo-red staining which indicated that the xylanase was active (supplementary data).

### Mass-spectrometry and Sequence analysis

Mass spectrometry data showed 228 amino acids with 2 unique peptides of protein (supplementary data). The molecular weight and isoelectric point of the protein were estimated to be 24.5 kDa and 6.29 respectively. Putative proteins are identified using the bioinformatics tools while they exhibit sequence similarity to an extent with characterized proteins. Usually, the similarity is found only in their conserved amino acid residues and no other significant region is similar to annotated proteins [10]. The amino acid sequence similarity and molecular structure of the protein define the enzyme classification system. 133 different glycoside hydrolase families have been reported [http://www.cazy.org] to date. Enzymes within a specific family have similar three-dimensional structure [11] and similar molecular mechanism [12]. Xylanases have been classified to families 10 and 11 exclusively where family 11 contains monospecific xylanases.

### Homology modeling of Xylanase

**Fig. 4:**
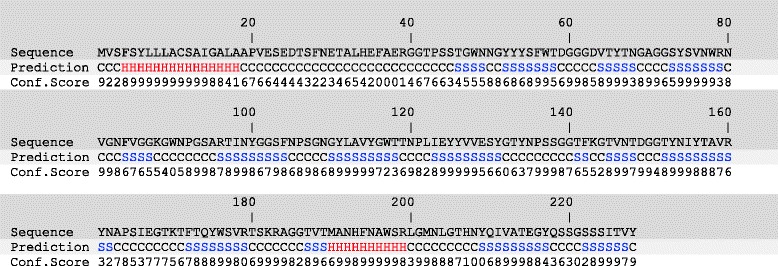
Secondary structure of Xylanase predicted by PSI-PRED. *Red* Alpha helix (); *blue* beta sheet () and *black* coil (C) whereas *Conf. Score* is the confidence score of each amino acid ranging from 1 to 9

To predict the secondary structure of the protein sequence of xylanase, profile-profile alignment was performed with 5798 non-redundant protein structures in I-TASSER (Iterative Threading ASSEmbly Refinement) employing PSI-BLAST (Position-Specific Iterative Basic Local Alignment Search Tool). The secondary structure was hence predicted using PSI-PRED. All α-helices, β-sheets and coiling were identified with their confidence scores in the predicted structure (Fig. 4). The query sequence was threaded through PDB library using LOMETS after the prediction of secondary structure by PSI-PRED. Initially to obtain the best performance from the ten state-of-art treading programs in LOMETS, top ten models were elected from each program solely based on sequence identity coverage and combined energy Z-score values (Table 2). Fold 2vgdA, the top ranked threading fold was identified by three individual threading programs in LOMETS with 66 % sequence identity of the templates in the threading alignment region with the query sequence. The threading folds were considered based on the Z-scores. More the Z-score value, more the significance level of the predicted structure. As it’s evident from the Table 2 that many folds have Z-score values, for instance 3zseA (5.63) or 1xndA (3.76) have more than the Z-score value of 2vgdA which creates ambiguity to predict the true positive structural fold of the protein. Hence, to identify the false positive matches and highlight true positive folds, another programming template modeling align (TM-align) was performed. The result of this structural alignment was ranked according to the TM-score (Table 3). Fold 2vgdA was thus identified and ranked in the first order (TM-score = 0.833; RMSD = 0.61). Hence, 2vgdA was identified as the possible native structure of the query sequence.

**Table 2:** Biochemical Characteristics. Accuracy refinement of predicted model 1 based on Z-score. Top ten treading folds were employed by I-TASSER to determine the structure of protein model

Mr	53.8 kDa
pI	5.27
Optimum pH	5
Optimum temperature	50^0^C
pH stability	7-9
Vmax	87.6 μmol mg^−1^ ml^−1^
Km	11.66 mg/ml

**Table 3:** Accuracy and refinement of structural analogs of predicted model 1 determined on the basis of TM-Score

Rank	TM-score^a^	RMSD^b^	IDEN^C^	Cov.^d^	PDB Hit
1	0.833	0.61	0.646	0.842	2vgdA
2	0.829	0.96	0.596	0.847	3b5lB
3	0.823	0.63	0.648	0.833	3wp3A
4	0.818	0.66	0.614	0.829	1xndA
5	0.817	0.69	0.688	0.829	1xnkA
6	0.805	0.86	0.652	0.820	3zseA
7	0.803	1.04	0.495	0.825	1h4gB
8	0.803	1.32	0.619	0.829	1pvxA
9	0.802	1.07	0.612	0.825	1ynaA
10	0.802	1.08	0.825	0.825	2dcjB

^a^Measure of structural similarity between predicted model and the native structure

^b^Root mean squared deviation between residues that are structurally aligned by TM-align

^c^Percentage sequence identity in the structurally aligned region

^d^Coverage of the alignment by TM-align = number of structurally aligned residues divided by length of the model

### Prediction of 3D structure

**Fig. 5:**
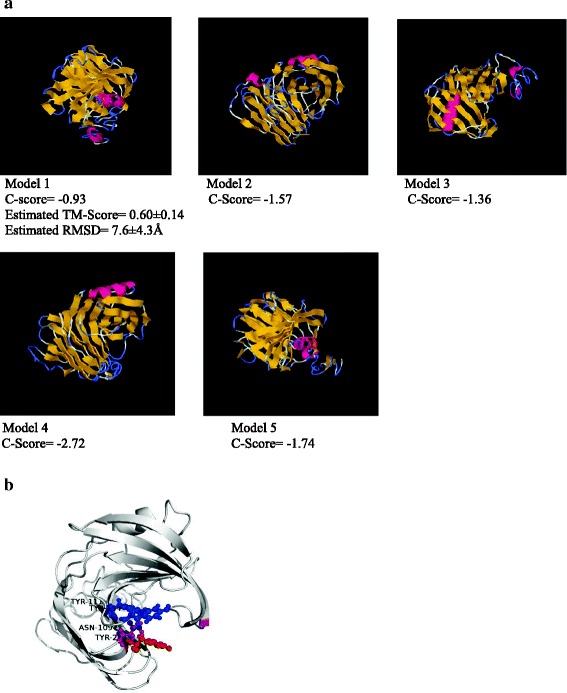
a Top five 3D models of the xylanase predicted by I-TASSER. Highest rank is marked by the lowest number of model (Model 1) by I-TASSER where as the highest number denotes the lowest ranking (Model 5). The ranking of the model was predicted by quality of the model which again was determined on the basis of C-Score, TM-score and RMSD. The C-scores of the models were determined based on the Z-score and the convergences of I-TASSER simulations as depicted in Table 2. b Final cartoon structure of Xylanase as derived from model 1 as shown in Fig. 5a. The substrate binding sites are Asn-109, Try-111, Tyr-134 and Tyr-217

Five top ranking 3D models were generated by the I-TASSER server by employing the top ten threading folds as the native structures (Fig. 5). Each model was validated based on C-score (Confidence-Score), TM-score, RMSD (The root-mean-square deviation) and cluster density. The model 1 had the highest *C*-score (-0.93) value reflecting a model of better validation with the structural similarity between the predicted model and the native structure (TM-score = 0.60 ± 0.14 and RMSD = 7.6 ± 4.3A^o^).

### Prediction of EC number and Ligand Binding site

Model 1 which has the highest C-score (−0.93) was then analyzed by some benchmarking tests on the I-TASSER server for potential enzyme analogs prediction. The analysis showed that the model 1 predicted by I-TASSER matched with five enzyme analogs, all of which exhibited identical EC nos.: 3.2.1.8 (Table 4) whereas the EC-scores (Enzyme Commission-Score) of those analogs differed. Highest EC-score was recorded in the PDB hit 2vgdA was 0.591 whereas the lowest was 0.582 for PDB hits 1hixB and 1te1B. As per ExPaSy enzyme database, the name of the protein analog 2vgdA is Endo-1,4-beta-xylanase whose biological function is endohydrolysis of 1,4-beta-D-xylosidic linkages in xylan. The ligand binding sites of the query protein were predicted using I-TASSER for further verification of protein function. This is based on determination of binding site scores (BS-score) which depend on the structural similarity and local sequence between the query structure and the binding sites of the templates. Based on the BS-score (more than 1.1 indicates prediction with high confidence) residues Asn-109, Tyr-111, Tyr-134 and Tyr-217 were identified as the active binding residues of the query protein (data not shown).

**Table 4:** Prediction of functional analogs of model 1 based on the enzyme classification (EC) score. EC-score is the confidence score for the EC number prediction determined using global and local structural alignment program. EC-score value range in between 0 and 1. Higher the EC-score, higher the confidence of EC number prediction

Rank	TM-score	RMSD	IDEN	Cov.	EC-score	PDB Hit	EC No.	Active Sites Residues
1	0.833	0.61	0.646	0.842	0.591	2vgdA	3.2.1.8	124, 215
2	0.821	0.71	0.684	0.833	0.588	1h1aA	3.2.1.8	124, 215
3	0.807	0.94	0.630	0.829	0.585	1enxA	3.2.1.8	124, 215
4	0.804	0.69	0.661	0.816	0.582	1hixB	3.2.1.8	124, 215
5	0.813	0.92	0.689	0.833	0.582	1te1B	3.2.1.8	124, 215

## Biochemical characterization of purified xylanase

### Effect of pH on xylanase activity

**Fig. 6:**
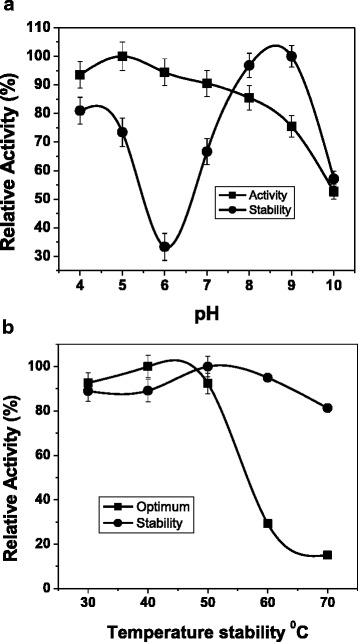
**a** Effect of pH on activity (■) and stability (▲) of xylanase from *Aspergillus fumigatus* R1. The effect of pH on xylanase activity was determined at 37^0^C for 30 min. where as the stability at 37^0^C after incubation for 1 h in various buffers. Buffers used: citrate (pH 4), acetate (pH 5), sodium-phosphate (pH 6-7), Tris (pH 8), glycine-NaOH (pH 9) and carbonate-bicarbonate (pH 10) (the results were obtained with triplicate sets of experiments). **b** Optimal temperature (■) and thermal stability (∆) of xylanase from *Aspergillus fumigatus* R1. The temperature profile was measured at different temperatures using optimum pH 5.0, 50 mM acetate buffer. For thermal stability, the enzyme was incubated at different temperatures (30-70^0^C) in acetate buffer (pH 5) for 1 h. (the results were obtained with triplicate sets of experiments)

The enzyme activity is greatly affected by pH. For the substrate binding and catalysis depend on charge distribution of both substrate and the enzyme molecules. The reaction pHs were adjusted to 4.0–10.0 with various buffers as described above. Xylanase was active over wide range of pH values from 4.0–10.0 with an optimum pH of 5.0; the optimal acetate buffer pH was 5.0 and retained more than 90 % of its activity (Fig. 6a).

### Effect of pH on xylanase stability

The stability of the purified xylanase from *Aspergillus fumigatus* R1 was determined at 37 ^0^C for 1 h when tested over a wide pH range of 4.0–10.0 (Fig. 6a). The xylanase was found to be highly stable at pH values of 8.0 and 9.0. The enzyme retained 70 % of its activity at pH 9.0 for 1 h at 37^0^C.

### Optimum temperature for xylanase activity

The xylanase was active over a broad temperature range of 40–60 ^0^C and showed its optimal temperature at 50^0^C (Fig. 6b).

### Thermal stability of xylanase

The enzyme was found to be more stable at temperature below 50^0^C. The enzyme retained about 40 % of its activity at 60^0^C after 30 min of incubation (Fig. 6b). Thermal stability of the enzyme is considered to be one of the significant characteristics in the industry; the thermostability of the enzyme can be increased by different methods like addition of metal ions or by protein engineering.

### Determination of kinetic parameters

**Fig. 7:**
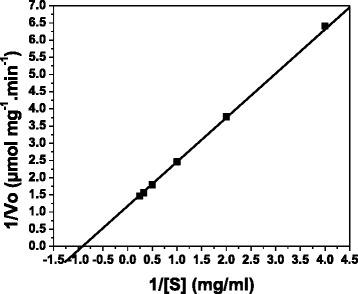
Lineweaver-Burk plot of initial velocity data for xylanase from *Aspergillus fumigatus* R1. The xylanase activity was determined in 50 mM acetate buffer pH 5.0 at 50 ^0^C. Symbols: *V*_*0*_ in μmol mg^-1^ min^-1^; [S]: birchwood xylan concentrations in mg ml^-1^

*Km* is the Michaelis constant, can be found by measuring the substrate concentration when half the max velocity is achieved. *Km* is a constant that remains same for a given set of enzyme and substrate. Therefore, low *Km* increases the affinity of enzyme with the substrate. The rate dependence of the enzyme reaction on different birchwood xylan concentrations at pH 5.0 and 50 ^0^C followed Michaelis-Menten reaction kinetics. Reciprocal plots (Fig. 7) displayed apparent *Km* and *Vmax* values of 11.66 mg ml^−1^ and 87.6 μmol min^−1^ mg^−1^. In this study, the purified enzyme showed significantly low *Km* towards birchwood xylan.

## Discussion

In spite of advanced knowledge of microbial xylanases in the past decades, several terms are still significantly considered for choosing a microorganism for the production of xylanases. Pulping industries require cost effective ways to produce xylanases which must have significant biochemical characteristics to withstand the hostile catalytic conditions. Agricultural residues serve as the prominent source of hemicelluloses which are both environment friendly and cost effective. In the present study, the xylanase has been produced using agricultural residue (rice bran) as the only source of carbon from *Aspergillus fumigatus* R1 which yielded 208U/ml of xylanase in 96 h of incubation. *Aspergillus* species have been the target organisms for the production of xylanases mainly because of the production capacity using the submerged fermentation as compared to the bacteria where the xylanase production is found to be low [8]. Xylanase production has been reported from many *Aspergillus* species. The xylanase activities of *Aspergillus fumigatus* Z5 [13] and *Aspergillus fumigatus* MA28 [14] had been found to be 15.02U/ml and 8.45U/ml respectively whereas the xylanase activity of *Aspergillus fumigatus* R1 was reported to be 208U/ml in 96 h of incubation. Thus, xylanase production by *Aspergillus fumigatus* R1 is found to be energy efficient and less time consuming which is an important criterion for selection of strain in industry for cost-effective production.

For *in silico* studies of the purified xylanase, the protein sequence containing 228 amino acids obtained after mass-spectrometry was analyzed by using the I-TASSER server [15]. I-TASSER protein structural modelling is based on the programme known as secondary structure enhanced Profile-Profile threading alignment. Secondary structure of the target protein was predicted by employing the I-TASSER and then a 3D structural model of the target protein indicating substrate binding residues was constructed. Nuclear membrane resonance (NMR) and/or protein crystallography have been used to construct 3D structural model of the target protein [16] but these techniques are more laborious, expensive and time consuming whereas, bioinformatics analyses offer comparatively less laborious, inexpensive and relatively faster experimentation. However, there are some shortcomings in these analyses; for instance, it is inevitably difficult to select most appropriate candidate when several variable parameters are to be considered in determination of structural models. In the current study, the threading fold 2vgdA (Z-Score 3.37) was selected as a native structure for further analysis of structural modelling (Table 2) where the other folds 3zseA and 1xndA shown higher Z-Score values (5.63 and 3.55 respectively Table 2). In this particular case the decision making factors were the sequence identities (Iden 1 and Iden 2). Thus, there is no problem in selection of a model when all required parameters are significantly higher. In this experiment, model 1 was selected as the top ranked model by I-TASSER which was based on the highest confidence score (C-score = -0.93; Fig. 5a) which was further validated by highest TM-score (0.60 ± 0.14) and RMSD (7.6 ± 4.3A^o^). Therefore, this model was selected for further studies. Hence, in the *in silico* studies the final model of purified xylanase predicted consists of residues Asn-109, Tyr-111, Tyr-134 and Tyr-217 which were identified as the active binding residues of the query protein.

Xylanase from *Aspergillus ficuum* AF-98 had optimum pH of 5.0 which retained 50 % and 30 % of its relative activities at pH 5.0 and pH 3.0 respectively [17]. The optimum pH of purified xylanase from *A. fumigatus* R1 is found to be close to other species of *Aspergillus* such as *A. kawachii* pH 5.5 [18], *A. nidulans* pH 6.0 [19], *A. foetidus* pH 5.3 [20]. The change in behaviour of the enzyme at pH 5 and 6 may be due to change in the composition of the buffer. The pH stability of xylanase between pH 7.0–9.0 (37^0^C) has been reported from other *Aspergillus* species such as *A. carneus* M34 [21] and xylanase stability at pH 2.0–11.0 (30^0^C), 5.2–5.7 (55^0^C) has also been investigated from different genera such as *Streptomyces olivaceoviridis* E-86 [22] and *Streptomyces olivaceoviridis* A1 [23] respectively. Thus, stability of the purified xylanase at broad pH range, especially to alkaline conditions is significant characteristic for applications in industry.

The optimum temperature of xylanase from *Aspergillus fumigatus* R1 was found to be similar to the xylanase from *Aspergillus fumigatus* MA28 [14] which showed optimal temperature of 50^0^C. The optimal temperature of purified xylanase from various strains of *Aspergillus* species is different such as *A. versicolor* (55^0^C) [24], *A. caespitosus* (50–55^0^C) [25], *A. ficuum* AF-98 (45^0^C) [17] and *Penicillium* species such as *Penicillium occitanis* Pol6 (45^0^C) [26]. The temperature stability of xylanase in the current study is similar to another xylanase from *Penicillium occitanis* Pol6 [26] which retained 50 % of its activity at 60^0^C after 1 h incubation. However, incubation at higher temperature decreased the xylanolytic activity. At 70^0^C, it lost 28 % of its activity in 30 min.

Kinetic parameter analysis of purified xylanase from *A. fumigatus* R1 showed that it had a *Km* of 11.66 mg ml^−1^ and *Vmax* of 87.6 μmol min^−1^ mg^−1^. Bakira et al. [27] studied the kinetic parameter of xylanase from *Rhizopus oryzae* and showed that *Km* value for xylanase towards birchwood xylan was 18.5 mg ml^−1^. The *Km* value of xylanase from *A. fumigatus* R1 was found to be similar to the *Km* value of xylanase from *Streptomyces cyaneus* SN 32 [28] which was found to be 11.1 mg ml^−1^ towards birchwood xylan. The *Km* values of xylanase II from *A. sydowii* SBS 45 [29] towards birchwood xylan and oat spelt xylan were reported to be 6.51 mg ml^−1^ and 7.69 mg ml^−1^ respectively whereas the *Vmax* of xylanase II for birchwood xylan and oat spelt xylan was found to be 1587 μmol min^−1^ mg^−1^ and 2381 μmol min^−1^ mg^−1^ respectively. *Penicillium occitanis* Pol6 [26] showed *Km* of 14.13 mg ml^−1^ and *Vmax* of 806.3 μmol min^−1^ mg^−1^_._

## Conclusion

In summary, we show that xylanase produced by *Aspergillus fumigatus* R1 under submerged fermentation is novel enzyme. The xylanase was analyzed by mass-spectrometry, which indicated that the xylanase under study has never been reported earlier. The purified xylanase showed acidic optimum pH and broad range of pH stability mainly in the alkaline region. These characteristics coupled with the xylanase production using agricultural residue as the only source of carbon make the enzyme suitable for many applications, especially in food industry and a probable alternative to the use of chemicals in bioleaching in paper and pulp industries. Taken together, we propose that the 3D model of xylanase established could be used for rational design of xylanase-specific inhibitors and to further understand the role of each fold of this protein to determine its function. Moreover, the predicted 3D structural model can be used as a basal structure for obtaining point mutation to improve the catalytic efficiency of this protein and may be to increase thermostability.

## Methods

### Materials

Birchwood xylan and dialysis tubing (M.W. cut off, 10 kDa; average flat width, 25 mm; capacity, 60 ml per foot) were purchased from Sigma (Sigma Aldrich Co. Ltd., Germany). Bio-Gel P-60 for gel filtration was purchased from Bio-Rad. Rice bran was obtained from a local mill. All other chemicals were of reagent grade unless otherwise mentioned and obtained from suppliers, HiMedia, Sigma, GeNei, Merck and SRL.

### Fungal strain isolation and culture conditions

*Aspergillus fumigatus* R1 was isolated in our laboratory from the soil samples (Puducherry, India) collected from the decaying paper and wood material. The strain was identified by lactophenol cotton blue staining and 18S rRNA sequence analysis. For isolation of xylanase producer, the preculture medium (g/L) contained: Birchwood xylan, 1; KH_2_PO_4_, 0.5; MgSO_4_, 0.25; NH_4_Cl, 1; Yeast-extract, 0.1. 1 g Soil sample was inoculated into the broth medium and incubated in shaker for 5–7 days at 70 rpm at 37 ^0^C. For xylanase production, the basal medium for the flask culture contained (g/L): Rice bran, 10; KH_2_PO_4_, 1; MgSO_4_.7H_2_O, 0.2; K_2_HPO_4_, 1.2; (NH_4_)_2_SO_4_, 5; CaCl_2_.2H_2_O, 0.03; FeSO_4_.7H_2_O, 0.01. Shake flasks, prepared each containing liquid basal medium, were inoculated with the fungal spores and incubated at 37^0^C.70 rpm for 96 h.

### Screening—Qualitative test

Culture from pre-culture medium was spread inoculated on sterile xylan-agar medium and incubated at 37^0^C. After 48 h, the plate was flooded with 0.1 % (*w/v*) Congo-red for 15 min followed by 10 min treatment with 1 M NaCl and subsequently applied with 5 % (*v/v*) acetic acid [30].

### Phylogenetic analysis

The 18S rRNA partial sequence of the isolate was compared with other closely related strains using BLAST and NCBI GeneBank data base. Alignment and the phylogenetic tree were constructed using MEGA 5.05 software [31]. The neighbor-joining (NJ) tree of the *Aspergillus fumigatus* R1 xylanase was evaluated using 1000 bootstrap replications [32]. The nucleotide sequence of the culture *Aspergillus fumigatus* R1 was submitted in NCBI GeneBank database with an accession number KJ001801.

### Xylanase production kinetics

The fungal culture was grown in basal medium containing rice-bran as the only source of carbon at 37^0^C for 24 to 96 h. At regular time lapse of 24 h, the samples were withdrawn under aseptic conditions to check the enzyme activity.

### Enzyme assay

Xylanase activity was assayed according to Bailey et al. [33]. The reaction mixture contained equal volumes of 1 % Birchwood xylan and the suitably diluted enzyme solution with 50 mM phosphate buffer (pH 7) at 37^0^C for 30 min. The amount of reducing sugar liberated was determined by Dinitrosalicylic acid method using xylose as the standard. One unit of the xylanase activity was defined as the amount of enzyme that catalyzes the release of 1millimol of xylose equivalent in 1 min. under standard assay conditions.

### Protein estimation

The protein concentration was determined according to the method of Lowry et al. [34] using bovine serum albumin as a standard.

### Purification of xylanase

All purification steps were performed at 16 ^0^C unless otherwise mentioned. The crude extracellular xylanase was obtained by centrifuging the culture broth at 8000 rpm for 10 min. The crude supernatant was subjected to 30–55 % fractional ammonium salt precipitation, stirred for 2–3 h and centrifuged at 10000 rpm for 10 min at 4 ^0^C. The pellet obtained was subjected to extensive dialysis for removal of salts. The dialyzed sample was further concentrated by lyophilisation. The lyophilized dialyzed sample, 1 ml, was applied to Bio-Gel P-60 gel-filtration column (5 cm × 52 cm). The column was equilibrated with 50 mM phosphate buffer (pH 7) and then washed with the same buffer. The packing of column was confirmed by constant flow rate of 0.2 ml/min. The void volume of the column was determined by blue dextran. Elution of proteins was carried out with the same buffer and *A*_280_ was used to monitor the protein in the purification steps. Fractions showing high specific activity were checked for their purity by SDS-PAGE. Those showing homogeneity of fractions were pooled and concentrated by lyophilisation and their purity and homogeneity was checked by sodium dodecyl sulphate-polyacrylamide gel electrophoresis (SDS-PAGE).

### SDS-polyacrylamide gel electrophoresis (SDS-PAGE) and activity analysis

SDS-PAGE was carried out with vertical slab unit (Genaxy scientific Pvt. Ltd., US) using 15 % (*w/v*) acrylamide in gels under denaturing conditions according to the method described by Laemmli [35]. The gels were stained by silver staining method [36]. The relative molecular mass (Mr) of the pure xylanase was determined by using protein molecular weight markers (GeNei PMW-L) as standard: (Ovalbumin 43 kDa, Carbonic Anhydrase 29 kDa, Trypsin Inhibitor 20 kDa, Lysozyme 14.3 kDa and Insulin 3.5 kDa). Activity analysis was performed under nondenaturating conditions using birchwood xylan with 1 % final concentration in 15 % (*w/v*) native PAGE according the method of Breccia et al. [37] after non-denaturing electrophoresis, the gel was incubated in acetate buffer pH 5 for 30 min and thereafter at 60 ^0^C for 15 min in the same buffer. Subsequently, the gel was stained with 0.1 % Congo-red to localize the band of protein with xylanase activity.

### Mass-spectrometric analysis

The purified xylanase band from native-PAGE was cut and sent to National Centre for Biological Sciences (NCBS), Bangalore, India for mass-spectrometric analysis. The purified protein was subjected to in-gel digestion with additional reduction and alkylation to bring in better sequence coverage according to Shevchenko et al. [38]. Digested peptides were reconstituted in 15 μL of the 0.1 % formic acid and 1 μL was injected onto agilent 1200 nano flow HPLC system in-line coupled though advion nanomate to orbitrap discovery. Peptides were separated on Agilent ZORBAX SB-C18 nano column by a gradient developed from 1 % [*v/v*] acetonitrile, 0.1 % [*v/v*] formic acid to 80 % [*v/v*] acetonitrile, 0.1 % [*v/v*] formic acid in water over 70 min at a flow rate of 300 nl/min. Full MS in a mass range between m/z 300–2,000 was performed in the Orbitrap mass analyzer with a resolution of 30,000 at m/z 400 and an AGC target of 5 × 10^5^. The strongest five signals were selected for CID (collision induced dissociation)-MS/MS in the LTQ ion trap at a normalized collision energy of 35 % using an AGC target of 3 × 10^4^ and two microscans. Dynamic exclusion was enabled with one count during 30 s and an exclusion period of 180 s. The exclusion mass width was set to 0.01. For protein/peptide identification MS/MS data was searched against both uniprot swissprot amino acid sequence database (non-redundant database with reviewed proteins) and Uniprot TrEMBL database (database with unreviewed proteins) downloaded in August 2013 using an in-house mascot server (version 2.4) through Proteome Discoverer 1.3 software. The search was set up for full tryptic peptides with a maximum of three missed cleavage sites. Carbamidomethyl on cysteine, and oxidized methionine were included as variable modifications. The precursor mass tolerance threshold was 10 ppm and the maximum fragment mass error was 0.8 Da. The significance threshold of the ion score was calculated based on a false discovery rate of < 1 % estimated by the peptide validator node of the Proteome Discoverer software. Minimum of two high confident peptides were used as a prerequisite to identify the proteins. 12.5fmoles of Standard BSA digest was analyzed at the beginning and end of sequence to check the performance of the instrument.

### Threading of protein structure

Two programs were employed on the I-TASSER server [15] to predict the secondary structure of the target protein xylanase: position specific iterated prediction (PSI-PRED) and position specific iterated-BLAST (PSI-BLAST). Initially, PSI-BLAST was performed for alignment of the query sequence against a non-redundant sequence database and then the secondary structure of the protein was predicted using PSI-PRED on the basis of the sequences generated by multiple alignments of the sequence homologs. LOMETS [39] is a locally installed meta-threading program on I-TASSER server which combines ten state-of-art treading programs (MUSTER, FFAS-3D, SPARKS-X, HHSEARCH2, HHSEARCH1, Neff-PPAS, HHSEARCH, pGenTHREADER, wdPPAS and cdPPAS). The secondary structure of the query sequence predicted was then threaded through a library of PDB structures using LOMETS. The templates were ranked on the basis of sequence-based profile-profile alignment, structure-based profile- profile alignment, secondary structure match, backbone torsion angle match, solvent accessibility match and general hydrobhobicity scoring matrix. Top-ranked template hits were selected from each threading program for further analysis.

### Assembly and Refinement of protein structure

Two programs were used Support Vector Machine SVMSEQ [40] and SPICKER [41] for assembly and refinement of the target protein structure. SPICKER is clustering program which performs clustering using representative set of decoy confirmations. Unified knowledge-based force field has three components which guide simulations of structural assembly: (1) PDB Cα/ side chain correlations [42], H-bonds and hydrobhobicity [43]; (2) spatial restraints of threading templates [44]; and (3) SVMSEQ sequence based contact predictions. There are two sets of simulations for determination of structural assembly. (1) Generation of initial structures for threading templates and (2) SPICKER generates cluster centroids from trajectories obtained in the first set of simulations. Finally, TM-align identifies the PDB structures which are structurally close to the cluster centroids [45].

### REMO: Modeling and 3D structure ranking for protein function prediction

The program REMO [46] depends on construction of full atomic models by optimizing H-bond network from C-alpha traces. Secondary structure specific backbone isomer was constructed using the program PSI-PRED. Full atomic model of our protein (xylanase) was constructed with reduced modeling simulation. Cα and side-chain centers play as the main simulation points for construction of models in reducing representation. These simulation points must be around to obtain better global topology of full atomic model with regular bond angle and bond length. In current study, Cα-RMSD and TM-scores parameters were considered for the verification of estimation of movement of Cα atoms relative to the native structure. Cα, C, N and O basic protein backbone fragments were matched from a secondary structure-specific backbone isomer library of non-redundant isomers from high-resolution PDB structures. H-bonding, clash/break-amendment, CHARMM22 [47] and I-TASSER were the driving parameters in REMO refinement protocol. CHARMM22 identifies the potential energy functions by mathematical equations and respective force field dimensions of biomolecules. The models constructed by I-TASSER were ranked on the basis of structural density in SPICKER clusters. Eventually, the 3D models which had the highest scores were compared with the proteins of known structure and function in the PDB. Structural analogs of query protein in enzyme commission (EC) numbers, substrate binding site libraries and active site residues were evaluated on the basis of global and local structural similarity.

## Biochemical characterisation of Xylanase

### Effect of pH on xylanase activity

The optimum pH for xylanase was determined under standard assay conditions with 1 % birchwood xylan dissolved in different 50 mM pH buffers, including citrate buffer (pH 4), acetate buffer (pH 5), sodium-phosphate buffer (pH 6–7), Tris buffer (pH 8), glycine- NaOH buffer (pH 9) and carbonate-bicarbonate buffer (pH 10). 0.786 μg of xylanase was incubated with 1 % xylan at different pH and the enzyme activity was carried out under standard assay conditions.

### Effect of pH on xylanase stability

For the pH stability test, 0.786 μg pure enzyme was placed in the above buffers with different pH and incubated at room temperature for 1 h and the relative activity was then assayed as described above at its optimum pH.

### Effect of temperature on xylanase activity

The optimum temperature for xylanase was determined under standard assay conditions, by incubating the enzyme at different temperatures (30–70 ^0^C) in 50 mM acetate buffer pH 5 (optimum pH) for 30 min and the relative activity was then assayed as described above.

### Effect of temperature on xylanase stability

To determine the temperature stability of xylanase, the enzyme was incubated at different temperatures (30–70 ^0^C) in acetate buffer (pH 5) for 1 h. The enzyme samples were cooled on ice for 10 min and relative xylanase activities were measured by the standard assay protocol as described above.

### Determination of kinetic parameters

Purified enzyme was incubated with various concentrations of birchwood xylan (2.5–40 mg/ml) in 50 mM acetate buffer (pH 5) at 50 ^0^C for 30 min. Kinetic parameters, *Km* and *Vmax* were calculated by linear regression from Lineweaver-Burk plots.

### Ethical approval and consent from patients/ participants

This work did not involve any studies on human volunteers.

## Abbreviations

BS-score: binding site-scores
CID: collision induced dissociation
C-score: Confidence-score
EC-scores: Enzyme Commission-score
HPLC: high performance liquid chromatography
I-TASSER: Iterative Threading ASSEmbly Refinement
LOMETS: local meta-threading-server
MS: mass-spectrometry
MW: molecular weight
PSI-BLAST: Position-Specific Iterative Basic Local Alignment Search Tool
RMSD: root-mean-square deviation
SDS-PAGE: sodium dodecyl sulphate-polyacrylamide gel electrophoresis
SVMSEQ: support vector machine
TM-align: Template Modeling align
